# Editorial: Vestibular migraine

**DOI:** 10.3389/fneur.2025.1587097

**Published:** 2025-03-21

**Authors:** Juan Manuel Espinosa-Sanchez, Chia-Cheng Lin

**Affiliations:** ^1^Division of Otoneurology, Department of Otolaryngology, Hospital Universitario Virgen de las Nieves, Granada, Spain; ^2^Otology and Neurotology Group CTS495, Instituto de Investigación Biosanitaria ibs.GRANADA, Granada, Spain; ^3^Division of Otolaryngology, Department of Surgery, University of Granada, Granada, Spain; ^4^Sensorineural Pathology Programme, Centro de Investigación Biomédica en Red en Enfermedades Raras (CIBERER), Madrid, Spain; ^5^Department of Physical Therapy, College of Allied Health Sciences, East Carolina University, Greenville, NC, United States

**Keywords:** vestibular migraine, dizziness, vestibular system, headache, migraine, episodic vertigo

Vestibular migraine (VM) is a disorder that occurs in patients with a previous or current history of migraine who experience recurrent episodes of vestibular symptoms with migraine features during these attacks. The International Bárány Society first published the diagnostic criteria for VM in 2012 (1) and updated them in 2021 (2). Although VM is underdiagnosed, it is considered the second most common cause of episodic vertigo after benign paroxysmal positional vertigo (BPPV), with a lifetime prevalence of about 3.2% and a 1-year prevalence of up to 2.7% in the general population (3–5).

The pathophysiology of VM is poorly understood, and several hypotheses have been proposed (6). It is likely that various mechanisms are involved, and both peripheral and central vestibular dysfunctions participate. In this sense, the study by Goto et al. included in this Research Topic reinforces the possible role of otolith dysfunction in VM pathophysiology by appreciating abnormal vestibular myogenic-evoked potentials (VEMP) responses and is aligned with some previous studies suggesting a vestibulospinal pathway involvement. On the other hand, VM and motion sickness are strongly linked, likely because of abnormal visuovestibular integration (7). Rova et al. have found that VM patients are significantly more motion-sensitive than non-VM patients. This finding supports the hypothesis that vestibular migraine is characterized by heightened motion sensitivity owing to abnormal multisensory processing (Rova et al.). Furthermore, Toriyama et al. have found that aura, tender point count (TPC), and interictal widespread pressure hyperalgesia (IWPH), rather than interictal allodynia, are more frequent in VM than in migraine with vestibular symptoms (MwVS) that fulfill the criteria for VM or migraine. The first finding supports the hypothesis that aura-related cortical spreading depression may contribute to transient vestibular dysfunction in VM, whereas TPC and IWPH indicate a state of enhanced pain processing in VM (Toriyama et al.).

Interestingly, VM is associated with significant multidomain cognitive impairment such that these patients show deficits in memory, executive function, attention, visuospatial abilities, and language (8, 9). In this Research Topic, Lu et al. have hypothesized that a dysfunction in frontal cortex circuits may explain this cognitive impairment. These authors have proposed the anti-saccade test, a measure of voluntary saccadic eye movement control, as a useful tool to evaluate and monitor cognitive decline in patients with VM (Lu et al.).

Vestibular migraine can present with spontaneous vertigo, positional vertigo, visually induced vertigo, or head motion-induced vertigo/dizziness with nausea in isolation or in any combination (2). These vestibular symptoms can occur before, during, or after migraine attacks. Auditory symptoms may appear in over two-thirds of patients (10). Rova et al. demonstrated that VM patients have a significantly higher frequency of a history of motion sensitivity than other vestibular patients. Even so, most vestibular patients, including those with VM, report no dizziness while driving (Rova et al.). Nevertheless, dizziness while reading as a passenger in a moving vehicle is strongly associated with VM (Rova et al.).

Clinical neuro-otological examination is often normal during the interictal period; nevertheless, mild ocular motor abnormalities such as saccadic smooth pursuit are often found between episodes. During an attack, low-velocity spontaneous and positional nystagmus, mostly of the central type, is common.

Vestibular function tests may reveal subtle oculomotor disturbances, although most patients exhibit normal video head impulse test (vHIT) and caloric testing results (2). VEMP results appear contradictory in the literature, with some reporting reduced or absent VEMP, while others reported no difference compared to controls (11, 12). Goto et al. have found that 32% (8/25) of patients with VM who underwent cervical VEMP (cVEMP) tests showed abnormal results, whereas ocular VEMP (oVEMP) were absent in 80% (20/25; six unilateral, and 14 bilateral).

There are no specific examinations, tests, or biological markers for VM; therefore, the diagnosis is made based on clinical history. Remarkably, the mere concurrence of vertigo and migraine is insufficient for diagnosis. Most patients with migraine and episodic vertigo do not have VM. The diagnostic criteria developed by the Barany Society and International Headache Society (IHS) are now widely accepted and have proven to be highly valid (1, 2). A thorough otoneurologic examination, functional tests, and imaging techniques may help exclude other conditions.

VM is a chameleonic disorder. The differential diagnoses include Meniere's disease (MD), BPPV, transient ischemic attacks, episodic ataxia type 2, and superior canal dehiscence syndrome. The overlap with MD makes the distinction between MD and VM a major diagnostic challenge, particularly in the first few years of the disease. Migraine and MD can be comorbid conditions; equally MD and VM can also coexist in the same patient according to the current diagnostic criteria (13).

Furthermore, VM is among the most common triggers for development of persistent postural-perceptual dizziness (PPPD) (14). Moreover, prolonged VM symptoms may transition to or overlap with those of PPPD. Similarly, some authors have suggested broadening VM diagnostic criteria to include chronic variants (15, 16). Rova et al. have found that patients with VM reported a history of motion sickness, which was significantly higher than that in patients with VM plus PPPD or PPPD alone.

VM treatment is categorized as acute or preventive. Patients often require symptomatic treatment during an acute episode, usually drug therapy; nevertheless, the evidence is very low (17). Depending on the impact on patients' quality of life, prophylactic treatment may also be required to reduce the frequency, duration, and severity of symptoms. Preventive treatments include dietary changes, lifestyle modifications, pharmacotherapy, and vestibular rehabilitation (17). Evidence for non-pharmacological interventions is also of low or very low certainty (17). Similarly, there is very limited evidence of drug therapy for prophylaxis (16). For this reason, from a practical point of view, pharmacological treatment of MV is based on the effectiveness of various drugs in the treatment of migraine (16). Vestibular rehabilitation may be useful for patients with motion intolerance and visual dependence (18). According to Goto et al., a better prognosis was observed in patients with bilateral oVEMP or asymmetry ratios of < 40 for cVEMP.

More research is needed to understand the pathophysiology, diagnosis, and treatment of VM, particularly through double-blind, placebo-controlled, randomized clinical trials.

## References

[1] Lempert T Olesen J Furman J Waterston J Seemungal B Carey J. Vestibular migraine: diagnostic criteria. J Vestib Res. (2012) 22:167–72. 10.3233/ves-2012-0453 23142830

[2] Lempert T Olesen J Furman J Waterston J Seemungal B Carey J. Vestibular migraine: diagnostic criteria1. J Vestib Res. (2022) 32:1–6. 10.3233/ves-201644 34719447 PMC9249276

[3] Lempert T Neuhauser H. Epidemiology of vertigo, migraine and vestibular migraine. J Neurol. (2009) 256:333–8. 10.1007/s00415-009-0149-2 19225823

[4] Neuhauser HK. The epidemiology of dizziness and vertigo. Handb Clin Neurol. (2016) 137:67–82. 10.1016/b978-0-444-63437-5.00005-4 27638063

[5] Formeister EJ Rizk HG Kohn MA Sharon JD. The epidemiology of vestibular migraine: a population-based survey study. Otol Neurotol. (2018) 39:1037–44. 10.1097/mao.0000000000001900 30020261

[6] Espinosa-Sanchez JM Lopez-Escamez JA. New insights into pathophysiology of vestibular migraine. Front Neurol. (2015) 6:12. 10.3389/fneur.2015.00012 25705201 PMC4319397

[7] Bednarczuk NF Bonsu A Ortega MC Fluri A-S Chan J Rust H. Abnormal visuo-vestibular interactions in vestibular migraine: a cross sectional study. Brain. (2019) 142:606–16. 10.1093/brain/awy355 30759189 PMC6391603

[8] Wang N Huang HL Zhou H Yu CY. Cognitive impairment and quality of life in patients with migraine-associated vertigo. Eur Rev Med Pharmacol Sci. (2016) 20:4913–7. 27981543

[9] Preysner TA Gardi AZ Ahmad S Sharon JD. Vestibular migraine: cognitive dysfunction, mobility, falls. Otol Neurotol. (2022) 43:1216–21. 10.1097/mao.0000000000003700 36136612

[10] Shi S Wang D Ren T Wang W. Auditory manifestations of vestibular migraine. Front Neurol. (2022) 13:944001. 10.3389/fneur.2022.944001 35911900 PMC9334870

[11] Baier B Stieber N Dieterich M. Vestibular-evoked myogenic potentials in vestibular migraine. J Neurol. (2009) 256:1447–54. 10.1007/s00415-009-5132-4 19377861

[12] Sanitha AS Sinha SK. Cervical and ocular vestibular-evoked myogenic potentials test results and its relation to vestibular signs and symptoms in individuals with vestibular migraine. Egypt J Otolaryngol. (2024) 40:63. 10.1186/s43163-024-00610-8

[13] Chen JY Guo ZQ Wang J Liu D Tian E Guo JQ. Vestibular migraine or Meniere's disease: a diagnostic dilemma. J Neurol. (2023) 270:1955–68. 10.1007/s00415-022-11532-x 36562849 PMC10025214

[14] Eggers SDZ Staab JP. Vestibular migraine and persistent postural perceptual dizziness. Handb Clin Neurol. (2024) 199:389–411. 10.1016/b978-0-12-823357-3.00028-8 38307659

[15] Tarnutzer AA Kaski D. What's in a name? Chronic vestibular migraine or persistent postural perceptual dizziness? Brain Sci. (2023) 13:1692. 10.3390/brainsci13121692 38137140 PMC10741489

[16] Webster KE Dor A Galbraith K Haj Kassem L Harrington-Benton NA Judd O. Pharmacological interventions for acute attacks of vestibular migraine. Cochrane Database Syst Rev. (2023) 4:Cd015322. 10.1002/14651858.CD015322.pub2 37042545 PMC10097606

[17] Webster KE Dor A Galbraith K Haj Kassem L Harrington-Benton NA Judd O. Non-pharmacological interventions for prophylaxis of vestibular migraine. Cochrane Database Syst Rev. (2023) 4:Cd015321. 10.1002/14651858.CD015321.pub2 37042522 PMC10091802

[18] Alghadir AH Anwer S. Effects of vestibular rehabilitation in the management of a vestibular migraine: a review. Front Neurol. (2018) 9:440. 10.3389/fneur.2018.00440 29946294 PMC6005864

